# Heart Rate Variability Measured from Wearable Devices as a Marker of Disease Severity in Tetanus

**DOI:** 10.4269/ajtmh.23-0531

**Published:** 2023-11-20

**Authors:** Ho Bich Hai, Jonathan W. S. Cattrall, Nguyen Van Hao, Hoang Minh Tu Van, Duong Bich Thuy, Phung Tran Huy Nhat, Phan Nguyen Quoc Khanh, Ha Thi Hai Duong, Tran Duc Duong, Ping Lu, Le Thanh Phuong, Heloise Greeff, Tingting Zhu, Lam Minh Yen, David Clifton, C. Louise Thwaites

**Affiliations:** ^1^ Oxford University Clinical Research Unit, Ho Chi Minh City, Vietnam;; ^2^ Department of Psychiatry, University of Oxford, United Kingdom;; ^3^ Hospital for Tropical Diseases, Ho Chi Minh City, Vietnam;; ^4^ University Medicine and Pharmacy, Ho Chi Minh City, Vietnam;; ^5^ Centre for Tropical Medicine and Global Health, University of Oxford, United Kingdom;; ^6^ Institute of Biomedical Engineering, University of Oxford, United Kingdom

## Abstract

Tetanus is a disease associated with significant morbidity and mortality. Heart rate variability (HRV) is an objective clinical marker with potential value in tetanus. This study aimed to investigate the use of wearable devices to collect HRV data and the relationship between HRV and tetanus severity. Data were collected from 110 patients admitted to the intensive care unit in a tertiary hospital in Vietnam. HRV indices were calculated from 5-minute segments of 24-hour electrocardiogram recordings collected using wearable devices. HRV was found to be inversely related to disease severity. The standard deviation of NN intervals and interquartile range of RR intervals (IRRR) were significantly associated with the presence of muscle spasms; low frequency (LF) and high frequency (HF) indices were significantly associated with severe respiratory compromise; and the standard deviation of differences between adjacent NN intervals, root mean square of successive differences between normal heartbeats, LF to HF ratio, total frequency power, and IRRR, were significantly associated with autonomic nervous system dysfunction. The findings support the potential value of HRV as a marker for tetanus severity, identifying specific indices associated with clinical severity thresholds. Data were recorded using wearable devices, demonstrating this approach in resource-limited settings where most tetanus occurs.

## INTRODUCTION

Tetanus is a severe neurological disease associated with significant morbidity and mortality, resulting from the effects of tetanus toxin within the central nervous system where it prevents motor and autonomic neuronal inhibition. Despite an effective vaccine, there remains a high global burden of disease, with most cases occurring in low- and middle-income countries (LMICs).^1^ The clinical syndrome is characterized by trismus and painful muscle spasms. In more severe cases, tetanus spasms lead to respiratory compromise and overt signs of autonomic nervous system dysfunction (ANSD), typically characterized by mixed sympathetic and parasympathetic signs of cardiovascular disturbance (fluctuating blood pressure, tachycardia), high temperature, sweating, and increased bronchial secretions.^2^

Increased catecholamines in urine and blood are associated with clinical signs of ANSD. Believed to result from disinhibition of the sympathetic nervous system, they are also predictive of disease severity and subsequent ANSD.^3^ For routine clinical practice, however, measurement is impractical, and alternative markers of disease severity are needed to inform clinical care decisions because in practice, ANSD may be difficult to distinguish from other causes of hyper- and hypotension or tachycardia. Heart rate variability (HRV) is the variability between consecutive heartbeats and reflects the neurocardiac axis, hence it indicates both sympathetic and parasympathetic nervous system activity.^4^ Conventionally measured from Holter electrocardiogram recordings, the advent of wearable devices makes this method suitable for use in low-resource settings and near real-time analysis suitable for clinical care.^5^

Preliminary data from a pilot study of 10 patients with severe tetanus showed significant differences in HRV parameters between patients with and without clinically apparent ANSD.^6^ More complex analysis using machine learning methods was able to distinguish ANSD achieving an F1 score performance of 86% and to classify severe or mild tetanus with an F1 score of 82%.^7^^,^^8^

Although these data are promising, limitations involve a lack of intepretability due to the “black-box” nature of machine-learning methods, the requirement for compatible bedside monitors to be available for data collection, and high computational costs for real-time monitoring. Our aim was to perform a large observational study to evaluate HRV in patients with tetanus, exploring HRV across the whole spectrum of tetanus disease, and to identify which HRV indices may be most relevant for clinical practice and understanding disease pathophysiology. We used wearable patch ECG devices for collection of data to establish the feasibility of these devices for data collection in an LMIC setting where most tetanus occurs and where bedside monitors with capacity for high-frequency data extraction are usually unaffordable. In this context, wearables connected to a mobile device would enable ECG reading and real-time monitoring of some HRV indices and would be an initial step toward a more complex machine-learning model approach to clinical guidance.

## MATERIALS AND METHODS

The study was carried out at the intensive care unit (ICU) of the Hospital of Tropical Diseases, Ho Chi Minh City, Vietnam, a 660-bed tertiary referral hospital that receives patients from southern Vietnam for the treatment of infectious diseases. Patients ≥ 16 years old between November 2018 and January 2020 were screened for eligibility. Those with a diagnosis of tetanus according to hospital guidelines within 48 hours of admission and no contraindication to wearable monitoring were eligible for enrollment.^9^ The study was approved by the ethical committees of the Hospital for Tropical Diseases and the Oxford Tropical Research Ethics Committee. All patients, or their representatives, gave written informed consent before participating in the study.

Patients were treated according to hospital guidelines. Spasms were treated with benzodiazepines as first-line agents, escalating to mechanical ventilation with nondepolarizing neuromuscular blocking agents.^10^ ANSD was treated with magnesium sulphate titrated against clinical effect and serum magnesium of < 4 mmol/L.

Clinical data were recorded prospectively and included routine clinical and demographic information. Tetanus severity was scored at all timepoints using the modified Ablett score (Grade 1 = no spasms; Grade 2 = spasms not interfering with respiration; Grade 3 = spasms interfering with breathing; and Grade 4 = Grade 3 + clinical signs of ANSD). Continuous ECG was recorded using wearable monitors (E-Patch Delta Electronics, Hörsholm, Denmark) for a period of approximately 24 hours after enrollment and 5 days later. The Day 5 point was chosen because we have previously shown that most patients with ANSD will have signs apparent by this point.^11^ ANSD was defined clinically by attending doctors according to previously used criteria with at least three of the following: heart rate > 100 beats per minute (bpm), systolic blood pressure > 140 mm Hg, mean arterial pressure < 60 mm Hg, pyrexia > 38°C, and fluctuating blood pressure occurring within 1 day with no other apparent cause.^6^

### ECG pre-processing and segmentation.

The ECG analysis was performed using the first three consecutive, stable, 5-minute sections from the 24-hour recordings after trimming of the first 10 minutes of recording. Segments then underwent adaptive filtering to remove abnormal RR intervals. An RR interval was considered abnormal if it deviated by > 10% from the mean length of the 100 preceding intervals. Additionally, intervals in which the heart rate was > 200 bpm, or < 45 bpm, were removed. RR interval detection was conducted using the Pan Tompkins algorithm.^12^ Frequency and time domains of each of the three, 5-minute sections, using the RHRV R package and the average of the three sections was calculated, resulting in eight time and six frequency domain indices.^13^^,^^14^ Analysis focused on these instead of nonlinear indices because they have well-described clinical correlates.^4^ Supplementary Table 1 describes a selection of HRV indices that have been extracted from the ECG waveform.

### Statistical analysis.

Our analyses focused on three key markers of disease severity: spasm, respiratory compromise, and ANSD. To compare participants with and without muscle spasms (i.e., Ablett score 1 versus Ablett score 2 and 3) and those with and without severe respiratory compromise (i.e., Ablett score 1 and 2 versus Ablett score 3), both Day 1 and Day 5 recordings were used. For those questions in which ANSD was not the clinical feature of interest, participants who were Ablett score 4 (i.e., clinical ANSD) were excluded from the analysis to ensure any differences in HRV identified between the comparator groups were not entirely secondary to the presence or absence of ANSD. To compare features between those with and without ANSD (i.e., Ablett score 1, 2, and 3 versus Ablett score 4), only Day 5 recordings were included because not enough participants had ANSD on Day 1 for statistical comparisons. The administration of either calcium channel blockers or magnesium sulphate was not included in the ANSD-related model due to insufficient occurrences of such events in both analysis groups. The period of onset was excluded in the spasm related models because it was intrinsic to class definition.

Wilcoxon rank sum tests were used to first compare differences in the median HRV indices for each of the groups of interest in an exploratory phase. Logistic regression was used to infer the association between HRV indices and severity classes. For datasets containing repeated measures on both Day 1 and Day 5, mixed-effects logistic regression was used to account for the participant random effect. Log transformation of indices was conducted before model estimation. The model included the aforementioned HRV indices in addition to age, sex, cardiac comorbidities (preexisting cardiovascular disease, or cardiac events before the day of HRV analysis), days from first symptom to admission, and administration of intrathecal antitoxin.

McFadden’s pseudo R^2^ value is reported for the multivariate models to infer if association between HRV and a particular severity exists. For mixed-effect logistic regression, the *R*^2^ value was calculated based on the conditional absolute deviance of the model and the deviance of a null model fitted without random effects. HRV indices display a high degree of collinearity (as shown in the Results). However, as each HRV index has clinical meaning that may hold some importance for the pathophysiological interpretation of the results, all HRV indices were included in the statistical analyses. Therefore, to test for coefficient significance in the model, in which HRV indices correlate with severity, the likelihood ratios test (Type II ANOVA) was used and a *P* value of 0.05 was taken to be significant. For the same reason, univariate logistic regressions were fitted for each HRV index to infer the direction of association between an HRV index and severity, based on which odd ratios are reported. R version 4.2.1 and packages of stats, gtsummary, car, and lme4 were used for all analyses.^15^

## RESULTS

One hundred ten patients were enrolled into the study between November 2018 and January 2020. HRV data collected on Day 1 were of satisfactory quality for extraction and analysis in 82 of 110 patients, and HRV data collected on Day 5 were of satisfactory quality for 80 of 110 patients.

Baseline characteristics of patients are shown in Table 1. Patients were predominantly male (91%), with a median age of 49 (interquartile range [IQR]: 42–60) years. Severity of tetanus varied, with 23 (21%) participants reaching maximum severity of Ablett 1, 25 (23%) Ablett 2, 37 (34%) Ablett 3, and 25 (23%) Ablett 4 by Day 5 of admission. Between Day 1 and Day 5, 16 (14.5%) participants improved (all from Ablett 2 to Ablett 1), 58 (52.8%) stayed unchanged, and 36 (32.7%) deteriorated (mostly by 1 Ablett point). Median length of hospital stay was 26 (IQR: 18–33) days.

**Table 1:** Baseline and clinical features of patients (*N* = 110)

Parameter	*n* (%) or median (IQR)
Sex	
Male	100 (91)
Female	10 (9.1)
Age (years)	49 (42, 60)
Preexisting cardiac disease	8 (7.3)*
Incubation period (days)	8 (6–14)
Unknown	21 (19)
Hospital stay (days)	26 (18–33)
ICU stay (days)	18 (9–23)
First symptom to admission (days)	3.5 (2.0–5.8)
Period of onset (hours)	48 (24–96)
No onset	3 (2.7)
Clinical outcome	
Discharged alive	103 (94)
Discharged to other facility	2 (1.8)
Palliative discharge	4 (3.6)
Died in hospital	1 (0.9)
Ablett score Day 1	
1	11 (10)
2	59 (54)
3	34 (31)
4	6 (5.5)
Ablett score Day 5	
1	23 (21)
2	25 (23)
3	37 (34)
4	25 (23)

ICU = intensive care unit; IQR = interquartile range.

* Observed on day 1.

**Figure 1.**
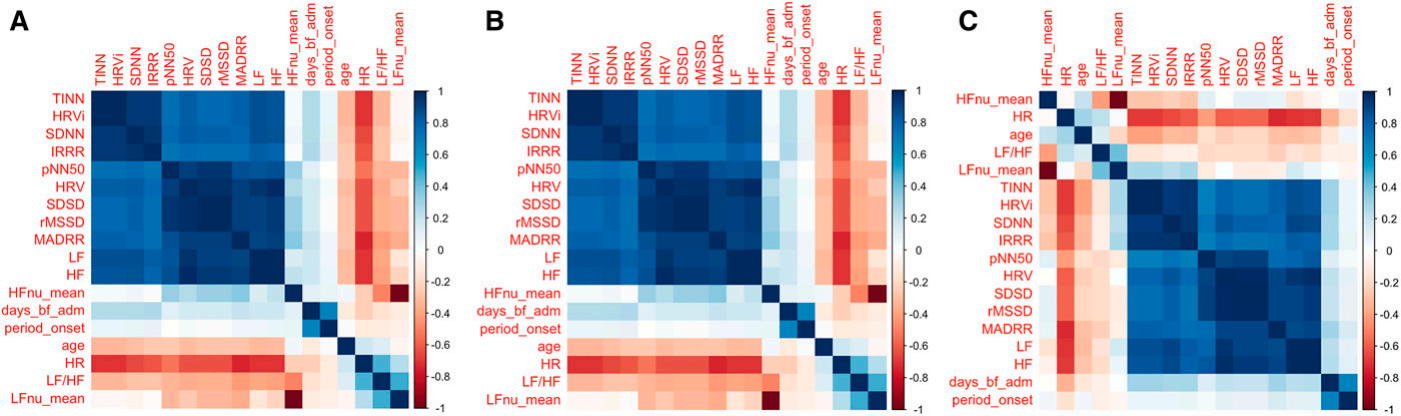
Correlation matrices of heart rate variability (HRV) indices. Correlation between HRV indices for (**A**) participants with and without muscle spasms, (**B**) with and without severe respiratory compromise, and (**C**) participants with and without autonomic nervous system dysfunction. Darker shading (red or blue) represents a greater correlation between the two indices. There is a high level of correlation between many of the indices in the dataset, justifying the use of the analysis of variance type II analysis.

HRV indices for the various comparisons are given in Supplementary Tables 2 through 4. Data show consistently lower HRV indices in more severe groups for each of the comparisons (i.e., spasms versus no spasms, respiratory compromise versus no respiratory compromise and ANSD versus no ANSD). Correlation matrices for these comparisons are shown in Figure 1, indicating, as expected, a high level of correlation between indices.

Results of significant HRV indices are shown in Tables 2 through 4 (models in Supplementary Tables 5–7). The clinical variable of time from first symptom to admission (3.5 [2.0–5.8] days) was an important variable in all three comparisons, with shorter periods associated with more severe disease. Different HRV variables featured in the different models. The IQR of RR intervals (IRRR) was the HRV variable most significantly associated with presence of muscle spasms compared with mild disease with no spasms (odds ratio [OR]: 0.99 [95% CI: 0.98–1.0]). For the next severity comparison, a decrease in low frequency (LF) (OR: 0.99 [95% CI: 0.98–1.0]) and high frequency (HF) (OR: 0.99 [95% CI: 0.97–1.0]) indices, were associated with the presence of severe respiratory compromise. (i.e., Ablett 3 compared with Ablett 1 or 2). The root-mean-square of successive differences between normal heartbeats (RMSSD), the standard deviation of differences between adjacent NN intervals (SDSD), the IRRR, the LF to HF ratio (LF/HF), and the total frequency power were significantly discriminative of ANSD versus non-ANSD. Decreases in RMSSD (OR: 0.98 [95% CI: 0.94–1.01]), SDSD (OR: 0.98 [95% CI: 0.94–1.01]), IRRR (OR: 0.93 [95% CI: 0.89–0.97]), and LF/HF (OR: 0.48 [95% CI: 0.11–1.26] were associated with the presence of ANSD.

**Table 2:** Significant heart rate variability indices associated with muscle spasms

Characteristic	OR	95% CI	*P* value
SDNN	0.99	0.97–1	0.08
IRRR	0.99	0.98–1	0.045
First symptom to admission	0.83	0.72–0.95	0.01
Timepoint	0.21 (Day 5)	0.06–0.71	0.005

IRRR = interquartile range of RR intervals; OR = odds ratio; SDNN = standard deviation of NN intervals.

CI is from mixed-effect univariate model. *P* value of likelihood ratio test from mixed-effect multivariate model with pseudo *R*^2^ 0.34.

**Table 3:** Significant heart rate variability indices associated with severe respiratory compromise

Characteristic	OR	95% CI	*P* value
LF	0.99	0.98–1	0.005
HF	0.99	0.97–1	0.004
First symptom to admission	0.36	0.15–0.83	0.01

HF = high frequency; LF = low frequency; OR = odds ratio.

*P* value of likelihood ratio test from mixed-effect multivariate model with pseudo *R*^2^ 0.83.

**Table 4:** Significant HRV indices associated with autonomic nervous system dysfunction

Characteristic	OR	95% CI	*P* value
SDSD	0.98	0.94–1.01	0.049
RMSSD	0.98	0.94–1.01	0.048
IRRR	0.93	0.89–0.97	0.049
LF/HF	0.48	0.11–1.26	0.007
HRV	1	0.99–1	0.004
First symptom to admission	0.6	0.37–0.85	0.055

HF = high frequency; HRV = heart rate variability; IRRR = interquartile range of RR intervals; LF = low frequency; OR = odds ratio; RMSSD = root-mean-square of successive differences; SDSD = standard deviation of differences between adjacent NN intervals.

CI is from univariate model. *P* value of likelihood ratio test from multivariate model with pseudo *R*^2^ 0.62.

## DISCUSSION

Our study has shown that collecting high-quality ECG data from ICU patients in a resource-limited setting is feasible. From these data, we have been able to calculate HRV indices and describe specific HRV features related to severity of tetanus.

Reduced HRV occurs in states of autonomic nervous system stress associated with acute and chronic illness and is a risk factor for cardiovascular events and mortality.^16^^–^^18^ Our previous pilot study of 10 patients with tetanus showed reduced HRV parameters in those with ANSD (Ablett 4) compared with those with severe disease but no ANSD (Ablett 3). Our current study confirms this result in a larger sample. Furthermore, our data show that there is an inverse relationship between severity and HRV across the whole spectrum of disease, not only limited to those with or without ANSD, demonstrated by a stepwise reduction in HRV with progressive increases in disease severity (moving from Ablett 1 to Ablett 4).

We note that specific HRV features were selected in the three models related to our research questions. Generally, HRV indices were least important as indicators of the mildest degree of tetanus (i.e., muscle spasms), indicated by the low pseudo *R*^2^ value of this model, and those that were selected were time domain features. In the other models evaluating parameters associated with more severe disease, in general HRV indices more specifically linked to the parasympathetic and sympathetic components of the autonomic nervous system were selected. In those with most severe disease, indices particularly linked to parasympathetic nervous system activity were selected as associated with clinically apparent ANSD. This might indicate increasing autonomic nervous system dysregulation with a relative reduction in the parasympathetic component occurring with increasing disease severity and is consistent with clinical features of hypertension, tachycardia, and observations of higher catecholamine concentrations in Ablett Grade 4.^3^^,^^11^ We have tried to correct for treatment factors in our models, but it should be noted that these differences may also be linked to treatment differences. Mechanical ventilation was not adjusted for, and although absent in Ablett Grades 1 and 2, it was consistent in those with Ablett Grades 3 and 4.

The pseudo *R*^2^ values indicate the likelihood of other explanatory variables existing, but not included, in our models. The inclusion of time from first symptom to admission in all three models demonstrates the importance of additional data. This variable is known to be an important predictor of outcome from tetanus in all age groups.^19^^,^^20^ A larger dataset would allow additional non-HRV data to be incorporated into models.

We have chosen 5-minute segments for comparison because these are standard for frequency domain variables but as such may have biased models against time domain variables that are ideally collected across the whole 24-hour period. The high degree of correlation between indices and the consistent patterns across all classes suggests that this effect is not so important, and all recordings were started during daytime working hours, reducing time-of-day bias.

Our methodology aimed to avoid excessive comparisons and make maximal use of data to answer our research questions. Consequently, for two of our questions, we combined data from Day 1 and Day 5. We included the day of recording as a variable in the models, including for spasms to address potential time point bias, and this was indeed a significant variable for spasm discrimination. As our analyses used only a short (5-minute) segment of ECG collected at the beginning of the 24-hour period, we may also be missing important changes occurring within the day of recording itself. Such longitudinal evaluation is more suited to machine-learning or deep-learning analysis, but this approach also requires high computational capacity, which limits its use for real-time clinical prediction. Furthermore, using a Day 5 reading may have missed some patients who subsequently developed ANSD, reducing the number of those with Ablett 4.

In conclusion, our study has demonstrated differences in HRV features related to disease severity in a large sample of patients with tetanus. These features can be calculated without the need for high computational power from short-duration ECG recordings obtained from wearable devices. We believe this not only advances our understanding of disease pathophysiology but indicates the feasibility of our approach as the basis for developing clinical decision support tools in the settings where most tetanus occurs.

## Supplemental files

10.4269/ajtmh.23-0531 Supplemental Materials
