# Supplementary material for: Heart Rate Variability Measured from Wearable Devices as a Marker of Disease Severity in Tetanus
Source: Am J Trop Med Hyg. 2023 Nov 20;110(1):165–9. doi: 10.4269/ajtmh.23-0531 (PMC10793034; doi:10.4269/ajtmh.23-0531)
Supplement: Supplemental Materials [file tpmd230531.SD1.pdf]

Supplementary Material

Supplementary Table 1: Heart rate variability indices

HRV index	Description
Time	
SDNN	Standard deviation of all NN intervals (ms)
pNN50	Number of pairs of adjacent NN intervals differing by more than 50ms in the entire recording divided by the total number of all NN intervals (%)
SDSD	Standard deviation of differences between adjacent NN intervals (ms)
rMSSD	Root mean square of successive differences between normal heartbeats (ms)
IRRR	Difference between third and first quartile of the RR intervals series (ms)
MADRR	Median of the absolute differences between adjacent RR intervals (ms)
TINN	Triangular Interpolation of the NN Interval Histogram (ms)
HTI	HRV Triangular Index
Frequency	
HF	Power in the high frequency range (ms^2)
LF	Power in the low frequency range (ms^2)
LF/HF ratio	Ratio of low frequency to high frequency
HRV	Total frequency power

Descriptions reproduced or adapted from Malik et al and Martínez et al. [13, 14]

Supplementary Table 2: Comparison of HRV indices: participants with and without muscle spasms

Muscle spasm	No (1), N = 26 [a]	Yes (2, 3), N = 116 [a]	p-value [b]
SDNN	48 (28, 78)	36 (22, 60)	0.052
pNN50	5 (1, 14)	2 (0, 11)	0.2
SDSD	28 (17, 38)	19 (11, 35)	0.056
rMSSD	28 (17, 38)	19 (11, 35)	0.056
IRRR	55 (36, 95)	49 (29, 77)	0.14
MADRR	14 (12, 22)	9 (5, 18)	0.014
TINN	148 (112, 202)	123 (86, 183)	0.072
HRVi	9.5 (7.1, 12.9)	7.9 (5.5, 11.7)	0.072
LF	36 (8, 76)	13 (4, 51)	0.029
HF	42 (11, 83)	16 (5, 63)	0.027
LF/HF	1.11 (0.86, 1.52)	1.22 (1.03, 1.47)	0.3
HR	77 (70, 87)	85 (71, 102)	0.042
HRV (Total power)	144 (46, 249)	68 (22, 195)	0.043

[a] Median (IQR); [b] Wilcoxon rank sum test

Supplementary Table 3: Comparison of HRV indices: participants with and without severe respiratory compromise

Severe resp. compromise	No (1, 2), N = 97 [a]	Yes (3), N = 45 [a]	p-value [b]
SDNN	45 (28, 69)	26 (14, 37)	<0.001
pNN50	5 (0, 15)	0 (0, 5)	<0.001
SDSD	24 (15, 40)	12 (9, 23)	<0.001
rMSSD	25 (15, 40)	12 (9, 23)	<0.001
IRRR	57 (36, 90)	35 (18, 56)	<0.001
MADRR	18 (8, 23)	7 (4, 12)	<0.001
TINN	144 (103, 205)	91 (63, 133)	<0.001
HRVi	9.2 (6.6, 13.1)	5.8 (4.1, 8.5)	<0.001
LF	22 (6, 64)	6 (2, 16)	<0.001
HF	31 (12, 85)	7 (3, 20)	<0.001
LF/HF	1.08 (0.90, 1.39)	1.38 (1.16, 1.50)	<0.001
HR	76 (67, 89)	99 (88, 114)	<0.001
HRV (Total power)	120 (46, 287)	25 (11, 83)	<0.001

[a] Median (IQR); [b] Wilcoxon rank sum test

Supplementary Table 4: Comparison of HRV indices: participants with and without ANSD

ANSD	No (1,2,3), N = 64 [a]	Yes (4), N = 16 [a]	p-value [b]
SDNN	36 (23, 61)	13 (8, 29)	<0.001
pNN50	2 (0, 9)	0 (0, 2)	0.041
SDSD	19 (10, 29)	8 (6, 15)	0.005
rMSSD	19 (10, 29)	8 (6, 15)	0.005
IRRR	51 (27, 78)	16 (11, 28)	<0.001
MADRR	8 (5, 15)	4 (4, 7)	<0.001
TINN	120 (85, 178)	53 (41, 81)	<0.001
HRVi	7.7 (5.4, 11.4)	3.4 (2.6, 5.2)	<0.001
LF	11 (4, 40)	2 (1, 5)	<0.001
HF	14 (5, 46)	2 (1, 6)	0.001
LF/HF	1.29 (1.06, 1.69)	1.15 (0.99, 1.56)	0.4
HR	89 (75, 104)	112 (100, 120)	<0.001
HRV (Total power)	48 (18, 158)	10 (5, 31)	0.002

[a] Median (IQR); [b] Wilcoxon rank sum test

Supplementary Table 5: Logistic regression estimation: participants with and without muscle spasms

Variable	Estimate	Standard error	Statistic [a]	p-value [b]
(Intercept)	-11.7	58.7	-0.2	0.842
**SDNN**	-3.9	2.3	-1.7	**0.087**
SDSD	2,409.5	4,118.5	0.6	0.559
pNN50	0.7	0.8	0.9	0.393
rMSSD	-2,411.2	4,120.9	-0.6	0.558
**IRRR**	5.2	2.6	2.0	**0.046**
MADRR	-2.5	1.6	-1.6	0.120
TINN	7.1	24.9	0.3	0.775
HRVi	-9.2	27.4	-0.3	0.738
LF	-4.2	3.1	-1.3	0.183
HF	2.9	4.4	0.7	0.503
LF/HF	0.3	0.2	1.2	0.249
HRV (Total power)	-0.1	4.7	0.0	0.991
HR	0.0	0.1	0.6	0.571
Sex (M)	0.6	1.4	0.5	0.647
Age	0.0	0.0	0.1	0.896
**Timepoint** (Day 5)	-1.8	0.7	-2.8	**0.005**
**First symptom to admission**	-0.2	0.1	-2.6	**0.010**
Cardiac comorbidity (Yes)	0.6	1.5	0.4	0.696
CCB or MgSO4	-1.6	1.2	-1.3	0.183
Intrathecal (Yes)	0.4	0.8	0.5	0.622
Patient random effect sd_(Intercept)	0.6			

[a] Statistic of intercept is z-score, statistics of coefficients are Chi-squared.
[b] Likelihood-ratio test

Supplementary Table 6: Logistic regression estimation: participants with and without severe repiratory compromise

Term	Estimate	Standard error	Statistic [a]	p-value [b]
(Intercept)	107.9	126.3	0.9	0.393
SDNN	-7.0	4.9	2.0	0.157
SDSD	-1,668.8	8,952.3	0.0	0.852
pNN50	-0.2	1.5	0.0	0.901
rMSSD	1,663.9	8,955.4	0.0	0.853
IRRR	3.0	4.3	0.5	0.478
MADRR	-0.8	2.4	0.1	0.748
TINN	-42.8	47.2	0.8	0.364
HRVi	50.5	54.5	0.9	0.355
**LF**	13.1	6.4	4.0	**0.046**
**HF**	-16.3	9.2	4.1	**0.044**
LF/HF	-0.1	0.3	0.1	0.702
HRV (Total power)	7.5	8.2	0.8	0.360
HR	0.0	10.5	0.0	0.998
Sex (M)	-0.2	2.4	0.0	0.931
Age	0.1	0.1	0.9	0.333
Timepoint (Day 5)	1.5	1.0	2.1	0.143
**First symptom to admission**	-0.9	0.4	6.5	**0.011**
Cardiac comorbidity (Yes)	-0.1	2.1	0.0	0.957
CCB or MgSO4 (Yes)	-0.9	1.8	0.3	0.588

Intrathecal (Yes)	0.6	1.5	0.1	0.712
sd_(Intercept)	2.7			

[a] Statistic of intercept is z-score, statistics of coefficients are Chi-squared.
[b] Likelihood-ratio test

Supplementary Table 7: Logistic regression estimation: participants with and without ANSD

Term	Estimate	Standard error	Statistic [a]	p-value [b]
(Intercept)	15.5	99.9	0.2	0.877
SDNN	12.3	9.9	2.1	0.152
pNN50	1.7	2.0	0.7	0.401
**SDSD**	-21,412.9	12,988.5	3.9	**0.049**
**rMSSD**	21,439.7	12,996.0	3.9	**0.049**
**IRRR**	-16.8	10.9	3.9	**0.049**
MADRR	2.3	2.7	0.7	0.392
TINN	7.1	45.2	0.0	0.874
HRVi	-6.5	54.5	0.0	0.904
LF	11.0	13.3	0.8	0.371
HF	-4.1	16.4	0.1	0.798
**LF/HF**	-15.7	9.1	7.4	**0.007**
**HRV** (Total power)	-19.6	9.7	8.4	**0.004**
HR	-0.1	0.1	0.7	0.387
Sex1	-4.7	3.3	2.5	0.110
Age	0.0	0.1	0.0	0.828
**First symptom to admission**	-0.8	0.5	3.7	**0.055**
Period of onset	0.0	0.0	0.6	0.426
Intrathecal (Yes)	-1.3	2.4	0.3	0.576
Cardiac comorbidity (Yes)	-0.3	1.5	0.0	0.826

[a] Statistic of intercept is z-score, statistics of coefficients are Chi-squared.
[b] Likelihood-ratio test
